# Second Language Accent Faking Ability Depends on Musical Abilities, Not on Working Memory

**DOI:** 10.3389/fpsyg.2019.00257

**Published:** 2019-02-12

**Authors:** Marion Coumel, Markus Christiner, Susanne Maria Reiterer

**Affiliations:** ^1^Department of Linguistics, University of Vienna, Vienna, Austria; ^2^Department of Psychology, University of Warwick, Coventry, United Kingdom; ^3^Department of Neurology, Section of Biomagnetism, University of Heidelberg Medical School, Heidelberg, Germany; ^4^Teacher Education Center, University of Vienna, Vienna, Austria

**Keywords:** phonological awareness, musical abilities, working memory, pronunciation ability, imitation task, accent faking

## Abstract

Studies involving direct language imitation tasks have shown that pronunciation ability is related to musical competence and working memory capacities. However, this type of task may measure individual differences in many different linguistic dimensions, other than just phonetic ones. The present study uses an indirect imitation task by asking participants to a fake a foreign accent in order to specifically target individual differences in phonetic abilities. Its aim is to investigate whether musical expertise and working memory capacities relate to phonological awareness (i.e., participants’ implicit knowledge about the phonological system of the target language and its structural properties at the segmental, suprasegmental, and phonotactic levels) as measured on this task. To this end, French native listeners (*N* = 36) graded how well German native imitators (*N* = 25) faked a French accent while speaking in German. The imitators also performed a musicality test, a self-assessment of their singing abilities and working memory tasks. The results indicate that the ability to fake a French accent correlates with singing ability and musical perceptual abilities, but not with working memory capacities. This suggests that heightened musical abilities may lead to an increased phonological awareness probably by providing participants with highly efficient memorization strategies and highly accurate long-term phonetic representations of foreign sounds. Comparison with data of previous studies shows that working memory could be implicated in the pronunciation learning process which direct imitation tasks target, whereas musical expertise influences both storing of knowledge and later retrieval here assessed via an indirect imitation task.

## Introduction

An analysis performed on the users of the language-learning app Busuu^1^ showed that 41% of them were learning a language to improve their career opportunities. Given that studies have shown that foreign-accented speech affects how listeners perceive their interlocutors (see the Accent Prestige Theory, Fuertes et al., 2002) and may even impact non-native speakers’ professional career (Hosoda and Stone-Romero, 2010), learning the accurate pronunciation of the target language seems fundamental to these learners. However, pronunciation training remains under-represented in foreign language teaching program (Gilbert, 2010) and research to improve pronunciation teaching methods are still needed. Fortunately, a plethora of studies is dedicated to the topic of foreign pronunciation learning and they can be split into two distinct categories. Much of the literature investigates ultimate second language (L2) attainment in bilinguals (early vs. late bilinguals) and tries to find out which speech features indicate learners’ non-nativeness (for instance, Flege et al., 1999; Moyer, 1999; Jilka, 2000; Weber, 2001; Winters and O’Brien, 2013; Sereno et al., 2015; Van Maastricht et al., 2018). A second category of studies seeks to identify which abilities support the acquisition of foreign language pronunciation. This category focuses on the dynamic process of language learning itself. Findings in this line of research revealed the existence of considerable individual differences in pronunciation learning abilities arising from the influence of various factors. For instance, working memory capacities (Papagno et al., 1991; Rota and Reiterer, 2009), empathy (Hu et al., 2012), mimicry ability (Reiterer et al., 2011; Hinton, 2013) and musical expertise (see for example, Slevc and Miyake, 2006; Oechslin et al., 2010) all constitute strong predictors of pronunciation learning abilities.

A commonly faced issue in the research on pronunciation acquisition is the difficulty to isolate individual differences in pronunciation abilities from the influences of other linguistic factors. The present study proposes to exploit the rarely used accent faking paradigm in order to specifically target pronunciation abilities. During the experiment, German native speakers had to fake a French accent while speaking in German. We then tried to see whether in such an experimental context, working memory and musical abilities remained strong predictors of individual differences in pronunciation abilities.

People highly vary in their capacity to reach native-like pronunciation of a foreign language (Golestani and Zatorre, 2009). This may arise from individual differences in the abilities to accurately perceive and to accurately produce foreign phonetic features and imitation tasks enable the simultaneous examination of both modalities. Indeed, in imitation tasks, participants hear a sentence and have to repeat it immediately after. Therefore, high performance on this task implies high capacity in both perception and production. Considerable individual differences exist in the capacity to imitate sounds, be it in a familiar foreign language such as English or in an unfamiliar foreign language such as Tamil or Hindi (Golestani et al., 2002; Reiterer et al., 2011, 2013). For example, results of neuroimaging studies which examined the neural correlates of pronunciation abilities showed that participants with lower speech imitation abilities evinced “cortical effort” (Reiterer et al., 2011, 2013), i.e., increased brain activations “in brain networks related to pronunciation, phonemic awareness, articulation, phonological processing, sound imitation, and auditory working memory” (Reiterer et al., 2013, p1). This suggests that imitation ability could be a source of individual differences in pronunciation abilities.

Direct imitation tasks suffer from several limitations. While imitating a second language they are familiar with, semantic or syntactic aspects of the sentences they have to repeat may distract imitators from focusing on the pronunciation itself. Similarly, while imitating a language they have never heard (Reiterer et al., 2013), imitators may be trying to reproduce the sequence of syllables rather than focusing on the prosody or the pronunciation of the sentences’ individual segments. On the contrary, a foreign accent faking task performed in subjects’ native language would require them to exploit only their phonetic and phonological knowledge of the target foreign language. Accent faking tasks specifically focus on participants’ phonological awareness, i.e., the knowledge of the target language’s phonological system they have acquired implicitly (Kivistö-de Souza, 2015).

Direct imitation tasks, because they mimic a learning mechanism which children exploit to acquire their first language (Deterding and Markham, 1999; Gathercole, 2006), constitute a learning task. In fact, mimicry ability appears to be a strong predictor of language learning aptitude (Purcell and Suter, 1980; Piske et al., 2001). To perform an accent faking task, on the contrary, learners need to retrieve phonetic representations they have built and stored before doing the task (Mora et al., 2013). This task type thus enables experimenters to assess intermediate achievement in foreign pronunciation rather than actual learning as in direct imitation tasks.

Uses of accent faking accent tasks are rare and usually serve the purpose of examining phonological awareness of specific features (Flege and Hammond, 1982; Zuengler, 1988; Mora et al., 2013). For instance, Flege and Hammond (1982) first introduced this paradigm by asking native speakers of English to read English items with a Spanish accent. Their aim was to determine potential differences in speakers’ perception of categorical versus non-categorical features. Mora et al. (2013) resorted to this task to investigate native Spanish speakers’ awareness of non-distinctive phonetic differences between English and Spanish. However, accent faking tasks seem adequate to measure previously acquired knowledge about the whole second language phonological system. There is only one study, to our knowledge, which adopted this angle of approach (Reiterer et al., 2013). In this experiment, German native speakers had to fake an English accent while reading German sentence. The authors observed that individual differences surfacing on the accent faking task correlated with performance on direct imitation tasks. This supports the idea that the two experimental designs rely at least partly on similar resources predicting pronunciation abilities. However, more investigations are necessary in order to identify the resources they may share. One possibility to fulfill this objective is to test whether the same factors predict performance on direct imitation tasks and on accent faking tasks. Since research has shown that both musical expertise and working memory capacities are strong predictors of pronunciation ability on direct imitation tasks (see subsequent paragraphs), we test whether they also predict individual differences in accent faking capacities.

Correlations between pronunciation abilities and music processing have long been established (Schön et al., 2004; Thompson et al., 2004; Pastuszek-Lipinska, 2008; Milovanov, 2009; Kraus and Chandrasekaran, 2010; Reiterer et al., 2011; Christiner, 2013) and music and speech perception may both improve with progresses in musical expertise (Schön et al., 2004; Magne et al., 2006; Oechslin et al., 2010). For instance, Slevc and Miyake (2006) recorded results of Japanese immigrants in the United States on three subtests of the Wing Measures of Musical Talent (Wing, 1968), a test measuring music perceptual abilities and contains a tonal memory production task. They found that participants with higher music abilities in both modalities displayed higher perceptual and productive phonological abilities. Along similar lines, musicians evince higher language imitation abilities than non-musicians (Nardo and Reiterer, 2009; Reiterer et al., 2011). Mimicry ability may actually be an element of musical aptitude (Gordon, 1989). Finally, a growing number of neuroimaging studies highlight the overlap between brain areas responsible for processing of language or speech and music (Özdemir et al., 2006; Rogalsky et al., 2011; Herdener et al., 2012; Merrill et al., 2012; Christiner and Reiterer, 2018). For example, the asymmetry observed in the planum temporale of musicians, a speech relevant area illustrates that musical training leads to neurological changes in brain regions relevant for language processing (Keenan et al., 2001; Luders et al., 2004).

Singing ability is a particularly strong predictor of pronunciation abilities (Nardo and Reiterer, 2009; Hu et al., 2012). When compared to musicians on a Hindi imitation task, vocalists evinced higher imitation scores although both groups obtained the same scores on the AMMA test (Advanced Measures of Music Audiation, Gordon, 1989) designed to assess perceptual musical abilities (Christiner and Reiterer, 2015) and musical aptitude (Schneider et al., 2002; Benner et al., 2017). The authors proposed that this pattern of results was due to increased vocal motor abilities in singers. In fact, learning to pronounce a new language, similarly to learning to sing, amounts to acquiring new motoric skills in the vocal apparatus (Hickok and Poeppel, 2007). Singing ability would thus enhance vocal flexibility and provide vocalists with the possibility to exploit a larger articulation space than non-singers (Reiterer et al., 2013). Within a few theoretical frameworks, increased vocal flexibility also leads to improvements in perceptual abilities. Speech perception means perceiving “intended gestures” in the motor theory of speech (Liberman and Mattingly, 1985); perception and production share representations relying on articulatory gestures according to Best’s hypothesis (1995). In other words, the fact that listeners perceive the gestural commands corresponding to the articulation of specific phonetic features may help them build shared representations for perception and production. Thus, singers, because they benefit from heightened vocal motor abilities could be able to both produce better sounds and store more accurate phoneme representations.

Performance on accent faking tasks depends on the developmental stage of participants’ oro-motor system (Reiterer et al., 2013). Indeed, the oro-motor system may be responsible for building memory of speech sounds (Schulze et al., 2012) and accent faking tasks require subjects to retrieve previously stored sound representations (Mora et al., 2013). Thus, musical abilities, by facilitating the development of the oro-motor system, could foster more efficient storing strategies of phonetic memories as well as better retrieval abilities. This would lead to high performance on accent faking tasks. This would be in line with previous studies which have shown that there is a link between musical expertise and higher memorization capacity (Nardo and Reiterer, 2009; Reiterer et al., 2011; Hu et al., 2012; Christiner and Reiterer, 2013).

In sum, because musical expertise and singing ability seem to relate to better speech perception and articulation capacity, we expect them to correlate with both direct imitation tasks and accent faking tasks.

According to Baddeley and Hitch (1974), working memory allows storage and processing of information over short time periods. It relies on three components: namely, a central executive system which controls the allocation of attentional resources and the coordination of information; and two components responsible for the storage of information, the visuospatial sketchpad and the phonological loop. Working memory and its subcomponents strongly relate to L2 learning outcomes (see Linck et al., 2014) and Miyake and Friedman (1998) even assimilate the whole language learning construct to working memory. Although a few studies did not find such a link (Hummel, 2002; Mizera, 2006), it seems that scores on tasks assessing the phonological loop predict L2 pronunciation learning (Papagno et al., 1991; Rota and Reiterer, 2009) and that L2 pronunciation abilities, fosters or is supported by working memory capacities (Rota and Reiterer, 2009). For instance, Papagno et al. (1991) observed that preventing articulatory rehearsal reduced learning capacity of unknown phonological forms. A reason for this could be that enhanced working memory capacities help L2 learners becoming aware of differences between first and second language phonological systems, facilitating thereby, the development of their phonological awareness (Mora et al., 2013).

Imitation tasks tax the phonological loop because this component is responsible for maintaining active verbal information over short periods of time. Such a task should indeed involve subvocal articulatory rehearsal mechanisms. Moreover, neuroscientific research suggests an overlap between the regions for phonological working memory and the areas subserving speech perception and production (Hickok and Poeppel, 2007; Acheson et al., 2011) which, as outlined above, imitation tasks combine. Along those lines, imitation abilities relate to scores on working memory tasks. For example, results on digit span and non-word repetition tasks correlated with Hindi imitation scores (Reiterer et al., 2011). The phenomenon of “cortical effort” observed in low ability imitators occurs in a premotor cluster responsible for the speech motor execution of articulatory movements as well as in the left inferior parietal lobule, an area subserving the phonological loop of auditory working memory (Reiterer et al., 2013). The experimenters interpreted this as an evidence that low ability participants had lower working memory capacities and needed to compensate for this disadvantage by an increase in activation in the brain regions supporting the phonological loop. Therefore, the available evidence suggests that imitation abilities depend on working memory, more precisely, on the phonological loop’s capacities.

Importantly, music competence may enhance working memory capacities. For instance, auditory working memory relates to musical abilities (Nardo and Reiterer, 2009; Christiner, 2013). Moreover, as neuroimaging studies show, the regions subserving short-term memory also support verbal and musical processing (Williamson et al., 2010; Schulze and Koelsch, 2012).

Because accent faking tasks rely on long-term memories (Mora et al., 2013; Reiterer et al., 2013), working memory capacities may not affect performance on this task. However, an indirect connection between working memory capacities and accent faking abilities may still exist. Indeed, the phonological loop supports L2 learning because it allows for the temporary storage of representations of new words and, through articulatory rehearsal, to engender their long-lasting storage/ the formation of long-term memories (Baddeley, 2003).

In sum, accent faking tasks allow to exclusively target pronunciation abilities and intermediate achievement in second language pronunciation since they assess previously stored phonetic knowledge. Thus, identifying the predictors of performance in faking accent allows assessing what influences retrieval and use of phonetic knowledge but does not allow for the identification of the predictors of pronunciation learning.

We conducted an exploratory study in order to investigate whether intermediate achievement in foreign pronunciation ability would be predicted by (1) musical perceptual and productive abilities;

(2) working memory capacities.

We expected accent faking abilities to correlate with both perceptual and productive musical expertise since this task requires participants to have a good articulation capacity (Reiterer et al., 2013). On the contrary, we anticipated that working memory capacities would not predict performance on the accent faking task because it taxes on long-term phonetic and phonological representations (Mora et al., 2013; Reiterer et al., 2013).

Such a pattern of results would allow drawing a distinction between resources that are required during learning of new phonetic and phonological material as opposed to the ones learners need in order to retrieve the knowledge after initial storage.

## Materials and Methods

To target individual differences in accent faking, we recruited German native speakers (*N* = 36) who pretended to be French native speakers by pronouncing German sentences with a French accent. Their recordings were rated by French native speakers. These listeners were instructed to determine the native language of the speakers they were listening to.

### Participants

The participants (*N* = 36) were students or young academics, German native speakers. The experimenters asked them to mimic a French accent. Participants’ age range was between 20 and 35. On average, the subjects were able to speak 2.51 foreign languages. Seventeen participants were male and eighteen female. Their first second language was English which was spoken by all of them. We recorded participants’ knowledge of French via two variables: French knowledge self-estimation and length of instruction in French. In the first case, subjects needed to rate from 1 to 5 their own proficiency in French, 5 being the maximum grade. In the second case, they had to report for how long they had received French instruction in years.

Participants read the following sentence sentence:

“Er hat seinem alten Vater damit keinem Gefallen getan.”

The participants read only one sentence for parsimony of testing design. The task was to be performed online and we wanted to make sure to keep it short enough for participants to persevere and complete the entire task.

All subjects gave their informed consent for inclusion before they participated in the study. The study was conducted in accordance with the Declaration of Helsinki and the protocol was approved by the Ethics Committee of the University Hospital and the Faculty of Medicine Tübingen, Project identification code 529/2009BO2.

### Ratings of French Accent

The recordings of the French accent faking task were rated by French native (*N* = 25) listeners. All of them were either coming from France or had been raised from birth in a French speaking environment. The ages ranged between 22 and 49 years. Listeners conducted this rating study online. We provided the instructions in French and asked the raters to listen to each sound file a maximum of four times in total. Finally, the listeners had to type in the language they thought was the mother tongue of the speaker. They were presented with three possible answers: “French,” “German” or “Other.” When choosing the “Other” option, they had to specify the language they were thinking about. Raters heard each sentence of the French faking task up to four times and these sentences were presented in a random order. In order to evaluate the performance of the participants’ French ability and to obtain a French score, we summed up how often each German participant was rated as being a French native speaker (i.e., a good “accent faker”).

### Musicality Measurements: AMMA and Singing Ability

Participants performed the AMMA test and self-reports of their singing ability. The AMMA test [Advanced Measures of Music Audiation (Gordon, 1989)] targets musical perceptual abilities whereby participants need to compare two musical statements and to judge whether they are identical or different. In the latter case, they additionally have to say whether the stimuli differ rhythmically or tonally. The entire test consists of thirty paired musical statements and is targeted at university music and non-music majors. The AMMA test is used to assess the concept of audiation, a process which involves that participants generalize and summarize musical statements which is different from imitation and memorisation. In the case of language, children learn to imitate language but not to memorize it and according to Gordon (1998), in music, audiation is what thinking is to language. Therefore, audiation is the ability to comprehend music which is no longer present.

To analyze the subjects’ singing ability, the participants performed a self-assessment of their singing ability. To this end, they answered two questions: (1) how well can you sing? (2) how much do you like to sing? by giving a grade between 1 (not at all) and 5 (very much). We decided to measure singing ability with self ratings because patterns of results obtained with this measurement method (Nardo and Reiterer, 2009; Reiterer et al., 2011; Hu et al., 2012) were replicated in experiments assessing singing ability on singing tasks and yielded comparable results to actual assessments done with singing recordings evaluated by expert or naïve singing teachers (Christiner and Reiterer, 2013, 2015, 2018) as long as non-professional and professional singers are mixed up in the research design. The self-estimation of professional singers is different from that of a layman. In this study, no professional singer was included.

### Working Memory

Participants did three phonological working memory tasks: a Wechsler digit span task (backward and forward) and a non-word repetition task. The first one is a subcomponent of a German version of the Wechsler Adult Intelligence Scale (Tewes, 1994). In this task, participants have to repeat in a forward or backward order a length-increasing sequence of numbers (from 3 to 9 numbers for the forward version and from 2 to 8 numbers for the backward version) presented auditorily. Participants have two chances per digit span and the test stops if they fail to repeat one of the two correctly. In the non-word repetition task, subjects need to repeat German monosyllabic non-words that were created from a syllable database developed according to German phonotactic rules (e.g., “knol,” “pflax,” “bamp”) at the Institute of Natural Language Processing, University of Stuttgart (Benner, 2005). Non-words were only tested in the forward condition because this task is more difficult than digit repetitions. Each string of non-words contains between 2 and 8 items and the item delivery is the same as in the usual digit span test. The participants’ working memory scores correspond to their accuracy of repetition on these three tests.

## Results

We adopted a relatively simple analytical design since we used a correlational approach to identify the markers of accent faking abilities.

### Descriptive Analysis

First of all, a high degree of inter-rater reliability was found on the accent faking task. The average measure ICC (intra-class correlation coefficient) was 0.932 with a 95% confidence interval from 0.899 to 0.958 [*F*(42,1050) = 15,236, *p* < 0.001]. In terms of distribution, among the French accent imitators, 4 participants were designated as being French native speakers by at least 20 raters (out of 25), 9 participants were designated as being French native speakers by at least 10 raters and the rest was identified as being French native speakers by less than 10 raters. 2 participants were rated as being native speakers of German by at least 20 raters and 1 participant was rated as being a native speaker of another language by at least 20 raters. In alphabetical order, the languages provided by raters as “other” mother tongues accumulate to a very interesting list: Arabic, Belgian, Chinese, Croatian, Danish, Dutch, English, Finnish, Greek, Hindi, Italian, Japanese, Norwegian, Persian, Polish, Romanian, Russian, Serbian, Spanish, Swahili, Swedish, Swiss, and Turkish. Among those, the most often cited languages were English (10%), Italian (5%), Spanish (4%), Chinese (3%), and Dutch (3%).

### Correlational Analysis

**FIGURE 1:**
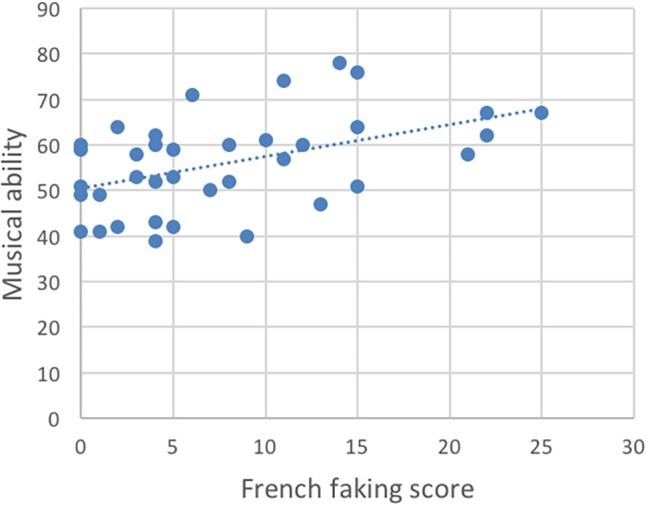
Correlation of speakers’ musical abilities on the two parts of the Advanced Measures of Music Audiation (AMMA) test with the French faking scores.

**FIGURE 2:**
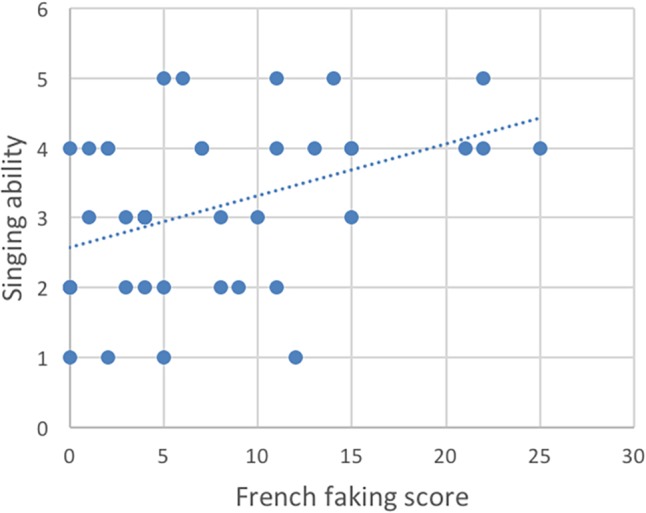
Correlation of speakers’ singing ability with the French faking scores.

**FIGURE 3:**
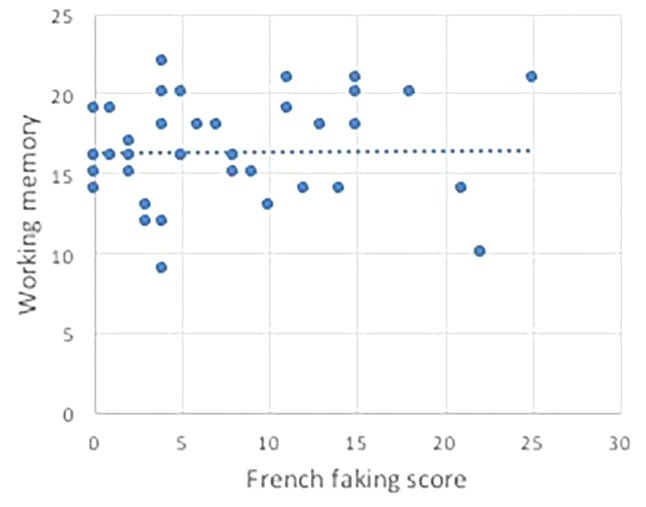
Absence of correlation between the working memory scores and the French faking score.

Table 1 shows the descriptive statistics. To analyze our data, we computed Spearman correlation coefficients to analyze how ratings on the French faking task related to the musical scores, working memory and French knowledge and length of instructions (Table 2). We found a significant relationship between the French faking scores and the tonal results on the AMMA test, *rs* = 0.39, *p* (one-tailed) < 0.05, the rhythmic part, *rs* = 0.40, *p* (one-tailed) < 0.01 (Figure 1), and answers to the question asking “how well can you sing?” *rs* = 0.40, *p* (one-tailed) < 0.01 and to the question asking “how much do you like to sing?” *rs* = 0.34, *p* (one-tailed) < 0.05 (Figure 2). There were no correlations to be found between the French scores and performance on working memory tasks (Figure 3). Thus, the pattern of correlations so far seems to corroborate our expectations. Finally, we observed that self-estimation of French knowledge scores correlated with accent faking scores [*rs* = 0.35, *p* (one-tailed) < 0.05], whereas the length of French instruction did not.

**Table 1:** Descriptive statistics regrouping all the variables.

Descriptive statistics				
	**M**	**SD**	**min**	**max**
French score	7.86	7.28	0.00	25.00
Singing ability	3.06	1.21	1.00	5.00
Singing like	3.69	1.16	1.00	5.00
Musicality tonal	25.94	5.42	16.00	37.00
Musicality rhythm	29.14	4.07	21.00	39.00
Musicality total	55.09	9.13	39.00	76.00
WM total	16.31	3.34	9.00	22.00
WM forward	8.89	2.41	4.00	14.00
WM backward	7.40	1.70	4.00	10.00
WM non-words	6.49	1.92	3.00	11.00
Self-estimation French	2.06	1.41	0.00	5.00
Instruction length	3.89	2.41	0.00	9.00

*M, mean; SD, standard deviation; Min, minimum score; Max, maximum score. French score = how often each speaker was rated as being a French native speaker, singing ability = answers to “how well can you sing?”, singing like = answers to “how much do you like to sing?”, musicality tonal = tonal perceptual abilities, musicality rhythm = rhythmic perceptual abilities, musicality total = average of “musicality tonal” and “musicality rhythm,” WM total = average of the three working memory tasks, WM forward = performance on the forward digit span task, WM backward = performance on the backward digit span task, WM non-words = performance on the non-words repetition task, self-estimation French = participant’s ratings of their own proficiency, instruction length = length of French instruction in years.*

**Table 2:** Results of the correlational analysis.

Correlations (Spearman)											
		French score	Singing ability	Singing like	Musicality tonal	Musicality rhythm	Musicality total	Working memory	Working memory forward	Working memory backward	Working memory non-words
French score	*r_s_*	1	0.40**	0.34*	0.39*	0.40**	0.41**	0.11	0.11	–0.06	0.18
Singing ability	*r_s_*	0.40**	1	0.73**	0.27	0.24	0.25	0.17	0.18	0.00	0.13
Singing like	*r_s_*	0.34*	0.73**	1	0.20	0.19	0.20	0.00	–0.08	0.01	0.24
Musicality tonal	*r_s_*	0.39*	0.27	0.20	1	0.83**	0.96**	0.10	0.03	0.15	0.26
Musicality rhythm	*r_s_*	0.40**	0.24	0.19	0.83**	1	0.94**	0.14	0.05	0.17	0.27
Musicality total	*r_s_*	0.41**	0.25	0.20	0.96**	0.94**	1	0.10	0.01	0.16	0.27
Working memory	*r_s_*	0.11	0.17	0.00	0.10	0.14	0.10	1	0.86**	0.73**	0.27
Working memory forward	*r_s_*	0.11	0.18	–0.08	0.03	0.05	0.01	0.86**	1	0.32*	0.16
Working memory backward	*r_s_*	–0.06	0.00	0.01	0.15	0.17	0.16	0.73**	0.32*	1	0.26
Working memory non-words	*r_s_*	0.18	0.13	0.24	0.26	0.27	0.27	0.27	0.16	0.26	1
Self-estimation French	*r_s_*	0.35*	0.38*	0.24	0.34*	0.16	0.24	0.00	–0.01	–0.07	0.23
Instruction length	*r_s_*	0.14	0.15	0.02	0.21	0.01	0.14	0.10	0.08	0.06	0.22

*^∗∗^Correlation is significant at the 0.01 level (1-tailed). ^∗^Correlation is significant at the 0.05 level (1-tailed).*

## Discussion

In a nutshell, the task was efficient in detecting individual differences in pronunciation ability since some participants were clearly better than others at faking a French accent. The correlational analysis showed that pronunciation achievement as measured on an accent faking task correlates with musical expertise but not with working memory capacities.

### The Contribution of Musical Expertise to Pronunciation Ability

The results of the present investigation corroborate that musical abilities, here music perception and singing ability, could account for individual differences in accent faking abilities. These correlations substantiate the previously reported links between individual differences in pronunciation ability and musical perception (Slevc and Miyake, 2006; Oechslin et al., 2010). In this investigation, the ability to detect rhythmic and tonal changes seems to be similarly related to faking French and higher musical perceptual abilities most likely improved the phonetic perception of the imitators and consequently allowed them to store more accurate memories of French segmental and prosodic aspects in long term memory, regardless of their length of French instruction. Indeed, length of French instruction did not influence French accent faking abilities. Moreover, these participants may have been able to store phonetic representations very efficiently based solely on exposure to French outside of school, for instance via the media. Along those lines, past research showed that musical expertise leads to “higher recall, memorization and imitation abilities” (Christiner and Reiterer, 2015; Fonseca-Mora et al., 2015).

Enhanced productive musical abilities also relate to high performance on the task as indicated by the relationship between French accent faking scores and the answers given to the singing ability self-assessment questionnaire. This is in line with research which identified singing ability as a strong predictor of imitation ability (Christiner and Reiterer, 2015) and with studies which found that heightened musical perceptual capacities do not suffice to account for better production (Christiner and Reiterer, 2013). First, high productive musical skills could support better speech production abilities via improvements in motoric skills. That is to say, acquiring enhanced motoric capacities in either singing or speech probably transfers to the other domain, or both singing and speech descend from a common source or “genetic ancestor.” Secondly, the ability to sing may provide vocalists with an increased awareness of the sound production process thus leading to enhanced musical perceptual capacities (Halwani et al., 2011). This relates to the Motor Theory of Speech according to which speech perception is perceiving “intended gestures” (Liberman and Mattingly, 1985). Singers should be better at discerning the articulatory gestures corresponding to the production of specific phonetic features which could allow them, in turn, to store more accurate memories of French phonemes. Actually, production and perception may build upon common representations (Best, 1995). Thus, the link between singing ability and performance on the French accent faking task could be that a high motoric flexibility developing with the capacity to sing helps forming phonetic representations of high quality that are common to perception and production. Motoric flexibility would then sustain the building of long term memories via the help of a well-developed oro-motor system (Schulze et al., 2012).

Since musical expertise relates to performance on both direct imitation tasks (Reiterer et al., 2011, 2013; Christiner and Reiterer, 2015) and accent faking tasks, musical abilities could be the pool of common resources these two task types share. In other words, musical expertise would be involved both in pronunciation learning and in intermediate achievement in pronunciation and mimicry ability is linked to musical competence regardless of the nature of the task.

### The Relationship Between Working Memory and Pronunciation Ability

There were no correlations between performance on the working memory tasks and results on the French accent faking task. At first glance, it could be surprising since the “phonological loop is used for short term retention of verbal information and is a necessary prerequisite for *later* imitation of verbal material” (Gathercole, 2000). Additionally, previous studies detected correlations between working memory capacities and participants’ imitation abilities measured on direct imitation tasks (Reiterer et al., 2011). However, faking an accent relies on long-term rather than on working memory capacities (Mora et al., 2013; Reiterer et al., 2013) whereas usual imitation tasks require high loads of working memory capacity (Christiner et al., 2018). For instance, working memory scores significantly correlated with performance on a Hindi direct imitation task, a language participants had never heard beforehand (Reiterer et al., 2011). Particularly, phonological working memory is a strong predictor of pronunciation aptitude in early but not in late learners (Hu et al., 2012). Our task did not require the participants to process new linguistic material since they were producing speech in their native language. On the contrary, subjects were tested on knowledge they had already acquired. This supports the idea that accent faking tasks inform us about intermediate achievement, rather than about learning itself. In other words, working memory seems not to be related to pronunciation ability itself but to its learning.

Measuring intermediate achievement enriches traditional approaches assessing learning of unfamiliar material. Indeed, these other approaches are concerned with potential and do not allow concluding about achievement. However, the present results do not discard the contribution of working memory to language learning. Rather, if the present task seemed not to tax this cognitive resource, initial storage of the invoked phonetic items most likely did. This would be in line with the fact that the role of working memory could be to transform transient phonetic events into rehearsable phonetic representations (Simmonds et al., 2011; Schulze and Koelsch, 2012) which in the present task participants would need to retrieve in order to mimic the target language.

Although music competence appears to enhance working memory capacities (Nardo and Reiterer, 2009; Hu et al., 2012), here, the fact that it correlated with accent faking abilities but not with working memory shows that both may be independent contributors to pronunciation ability (see also, Posedel et al., 2012). In other words, both musical perceptual and productive expertise and working memory may support initial storage of new linguistic material, while musical abilities but not working memory may assist retrieval of previously acquired knowledge. This is why working memory appears not to be a shared resource between direct imitation tasks and accent faking tasks.

### Limitation and Future Research

Limitations of the present study include the fact that we do not have information about participants’ exposure to French which did not result from an instructional context. This is problematic since some of the participants were able to fake a French accent but did not report having had French instruction. It suggests that these subjects were able to form phonetic and phonological representations via exposure to French in other contexts. It would be interesting to give participants a more detailed questionnaire in order to evaluate, which is the minimum amount of exposure to the target language necessary in order to gain reliable phonological awareness. In addition, multiple faking tasks performed in different languages should be introduced so that social distance, educational influence and aptitude for faking accents could be contrasted more precisely. Further research may also benefit from assessing subjects’ singing ability by having them sing during the experiment and their singing performance being rated by professional singers (see for instance, Christiner and Reiterer, 2015). This would allow to compare both tasks behaviourally.

## Conclusion

Our task showed that phonological awareness at intermediate achievement stages is linked to productive and perceptual musical abilities but not to working memory capacities. This leads to hypothesize that working memory could be recruited during learning (which may happen during direct imitation tasks), but not during retrieval and use of previously stored knowledge. Musical expertise, on the contrary, may advantage people both when storing new knowledge as well as when retrieving it on accent faking tasks for instance. Theoretically, the study provides some support for the idea that perception and production representations are shared. This would allow accounting for the link between musical and mimicry abilities and would explain the often-observed interaction between perception and production. Our results also show that individual differences in pronunciation abilities are not all due to variations in working memory.

This has both research- and pedagogically oriented consequences. First, accent faking tasks, which remain underexploited so far, can be used in order to assess (intermediate) achievement in learning foreign pronunciation. Moreover, this task seems to be useful to get rid of the influence of working memory and specifically target pronunciation abilities. Secondly, our results suggest that musical training should be included in language teaching in order to support learning of foreign language pronunciation and retrieval and later use of this acquired knowledge. Practicing singing, for instance, could facilitate second language pronunciation acquisition by increasing one’s awareness of sound production, by developing perceptual capacities and by supporting long-term storage of accurate foreign sound representations.

## Author’s Note

MCh is a recipient of a DOC-team-fellowship of the Austrian Academy of Sciences.

## Author Contributions

MCo and SR contributed to the conception and design of the study. MCo ran the experiments. MCo and MCh organized the database. MCh performed the statistical analysis. MCo wrote the first draft of the manuscript. MCh and SR wrote sections of the manuscript. All authors contributed to manuscript revision, read, and approved the submitted version.

## Conflict of Interest Statement

The authors declare that the research was conducted in the absence of any commercial or financial relationships that could be construed as a potential conflict of interest.

## Footnotes

1 Busuu survey: https://www.busuu.com/en/research/language-survey-2017/_05-text/busuu_language_learning_survey.pdf
